# miR-363-5p as potential prognostic marker for hepatocellular carcinoma indicated by weighted co-expression network analysis of miRNAs and mRNA

**DOI:** 10.1186/s12876-017-0637-2

**Published:** 2017-06-20

**Authors:** Jun Zhang, Jia Fan, Chongming Zhou, Yanyu Qi

**Affiliations:** Department of Oncology, The third people’s hospital of Chengdu, Chengdu, 610031 China

**Keywords:** Hepatocellular carcinoma, microRNA, Weighted co-expression network analysis, Prognosis

## Abstract

**Background:**

This study aimed to investigate potential miRNAs and genes associated with the prognosis of hepatocellular carcinoma (HCC).

**Methods:**

Weighted co-expression network analysis was utilized to analyze the mRNA and miRNA sequencing data of HCC from TCGA (The Cancer Genome Atlas) database. Significant network modules were identified, and then functions of genes in the gene network modules and target genes of miRNAs in the miRNA network modules were explored. Additionally, correlations between network modules and prognostic factors of HCC were analyzed.

**Results:**

In total, 10 mRNA network modules were identified, three of which were significantly related to tumor stage, NAFLD (non-alcoholic fatty liver disease) and patient age. Four miRNA network modules were identified, of which one was associated with tumor stage. Targets of hsa-miR-363-5p were found distributed in the gene network modules, such as RGPD5, RGPD6, ZNF445 and ZNF780B. Kaplan–Meier test revealed that low expression of hsa-miR-363-5p was associated with better overall survival of HCC patients.

**Conclusion:**

hsa-miR-363-5p may be a potential prognostic marker for HCC.

## Background

Hepatocellular carcinoma (HCC) is the most common primary liver malignancy with increasing incidence worldwide, which is mainly associated with chronic hepatitis B virus (HBV) and/or hepatitis C virus (HCV) infections, as well as alcohol consumption [1, 2]. There are few effective treatments for advanced HCC partly because the cell- and molecular-based mechanisms that contribute to the pathogenesis of this tumor type remain unclear.

Aberrant expression of microRNA (miRNA) has been detected in a variety of human malignancies and proved to be important influencing factors in cancer-associated genomic regions. Recently, remarkable studies have revealed the vital roles of miRNA in HCC pathogenesis. Several miRNAs are upregulated in HCC tissues compared to that in normal tissues, such as miR-21, miR-122, and miR-223 [3, 4], whereas some miRNAs were downregulated, such as miR-122a, miR-22 and miR-152 [5–7]. A series of miRNAs have been identified as tumor suppressors in HCC. For instance, the putative tumor suppressor miR-124 regulates cell aggressiveness of HCC by targeting ROCK2 and EZH2 [8]. miRNA-26a suppresses tumor growth and metastasis of HCC by modulating the interleukin-6-Stat3 pathway [9]. Overexpression of miR-101 blocks epithelial-mesenchymal transition and angiogenesis of HCC via decreasing multiple genes (e.g. COX2, EZH2 and STMN1) [10]. Furthermore, there are some studies identified the associations between several miRNAs and clinical outcomes of HCC patients. For instance, the high level of miR-425-3p is associated with time to progression and progression free survival [11]. Upregulation of miR-494 contributes to the lower survival rate of HCC patients [12]. However, there are still numerous of miRNAs and their targets that are associated with HCC prognosis remain to be identified.

Weighted gene co-expression network analysis (WGCNA) was used to explore the biological functions of genes based on RNA sequencing or microarray data in different samples [13]. In this study, in order to identify the potential key miRNAs and genes associated with the prognosis of HCC, weighted co-expression network analysis was performed on the mRNA and miRNA. The results may provide novel information for the study of HCC prognosis, and provide novel potential biomarkers for the clinical therapy of HCC.

## Methods

### Sequencing and clinical data

The clinical data of 377 patients with HCC were extracted from The Cancer Genome Atlas (TCGA, http://cancergenome.nih.gov/). Among the 377 patients, mRNA sequencing data for 371 patients and miRNA sequencing data for 372 patients of level 3 were available. Both mRNA and miRNA sequencing data were generated using the Illumina HiSeq platform..

### Data preprocessing

First, mRNAs and miRNAs with verbose <3 were removed using the goodSamplesGenes function in Weighted Gene Co-expression Network Analysis (WGCNA) (Version 1.43–10) package of R (Version 3.1.0) [14]. Meanwhile, abnormally low expressed mRNAs and miRNAs with RPKM (reads per kilobase of exon per million reads mapped) < 10 or deviation of average linkage distance and the main cluster >40% were removed, via an average linkage method. Subsequently, Pearson correlation coefficient between all of genes as well as miRNAs was calculated using the multiple testing, and the false positive rate < 5% was controlled by q-value [15].

### Weighted Gene co-expression network analysis (WGCNA)

Euclidean distance and Pearson’s correlation matrices were used to calculate the correlation between gene pairs and miRNA pairs, and the correlation between gene i and gene j (or miRNA i and miRNA j) was defined as s_i,j_ = |cor (μ_i_, μ_j_)|., where μi and μj represent expression value vector of i and j, respectively, and cor represents the Pearson correlation coefficient between the two expression value vectors. The calculated Pearson’s correlation matrices were transformed into matrices of connection strengths using a power function a_i,j_ = s_i,j_^β^. The β value is set as weighting coefficient only when the correlation coefficient between log(k) and log(p(k)) reaches 0.8, where p(k) represents the proportion of nodes with connectivity k. After the adjacency parameter was determined, the correlation matrix was transformed into an adjacency matrix, which was subsequently transformed into a topological overlap matrix. The topological overlap matrix (TOM) [16] was computed as follow:

$$ {\varpi}_{\mathrm{i}\mathrm{j}}=\frac{1_{\mathrm{i}\mathrm{j}}+{\alpha}_{\mathrm{i}\mathrm{j}}}{\left\{{k}_{\mathrm{i}},{k}_{\mathrm{j}}\right\}+1-{\alpha}_{\mathrm{i}\mathrm{j}}}, $$

where *1*_ij_ = ∑_*μ*_ *α*_i*μ*_ *α*_j*μ*_ indicates the product sums of the adjacency coefficients of the nodes connected to both i and j. *k*_↺_ = ∑_*μ*_ *α*_↺*μ*_indicates the sum of the adjacency coefficients of the nodes only connected to i. Similarly, *k*_j_ = ∑_*μ*_ *α*_j*μ*_ indicates the sum of the adjacency coefficient of the nodes only connected to j. If two nodes are neither connected each other nor share any neighbors, *ϖ*_ij_ = 0. The formula${d}_{\mathrm{ij}}^{\varpi}=1-{\upvarpi}_{ij}$ was used to calculate the dissimilarity degree between any two nodes.

### Identification of significant network modules

Genes were performed hierarchical clustering using the dissimilarity coefficient as the distance measure. Each branch corresponds to a module. Modules were identified by using a mixed dynamic TreeCut (Version 1.62) algorithm criterion [17]. The eigengenes in each module, which is stipulated as the first principal component of a given module and can be considered as a representative gene expression profile in a module, was calculated in turn when modules cannot be identified with the dynamic TreeCut algorithm criterion, then the merged close modules were clustered into new modules. The network significance approach determined the module related to prognostic factors based on module significance (MS). The MS indicated the average gene significance (GS) of all the genes in the module. Significant miRNA network modules were identified using the same methods as gene network module identification.

### Analysis of correlations between network modules and prognostic factors

Correlations between mRNA/miRNA network modules and seven prognostic factors of HCC [(gender, age, tumor stage, alcohol consumption, hepatitis B virus (HBV), hepatitis C virus (HCV) and non-alcoholic fatty liver disease (NAFLD)] were analyzed by calculating the Pearson correlation coefficient [18], and *p* < 0.01 was set as the cut-off criterion.

### Functional enrichment analysis of genes and miRNAs

Gene Ontology (GO) functional enrichment analysis of genes in the modules was conducted based on the Database for Annotation, Visualization and Integrated Discovery (DAVID, https://david.ncifcrf.gov/) [19, 20]. The *p*-value of each GO term was calculated by Fisher’s Exact Test [21], and only terms with *p*-value <0.05 were considered significant.

Furthermore, before GO enrichment analysis of miRNA targets, target genes of miRNAs in the modules were predicted based on the information in TargetScan (http://www.targetscan.org/), miRanda (http://www.microrna.org/microrna/home.do), and miRwalk (http://zmf.umm.uni-heidelberg.de/apps/zmf/mirwalk2/). Only the miRNA-gene pairs that were common in the three databases and met with false discovery rate (FDR) < 0.05 were chosen for further analysis. Afterwards, the target genes of miRNAs were underwent GO enrichment analysis, and only terms with *p*-value <0.05 were considered significant.

## Results

### Data preprocessing

**Fig. 1:**
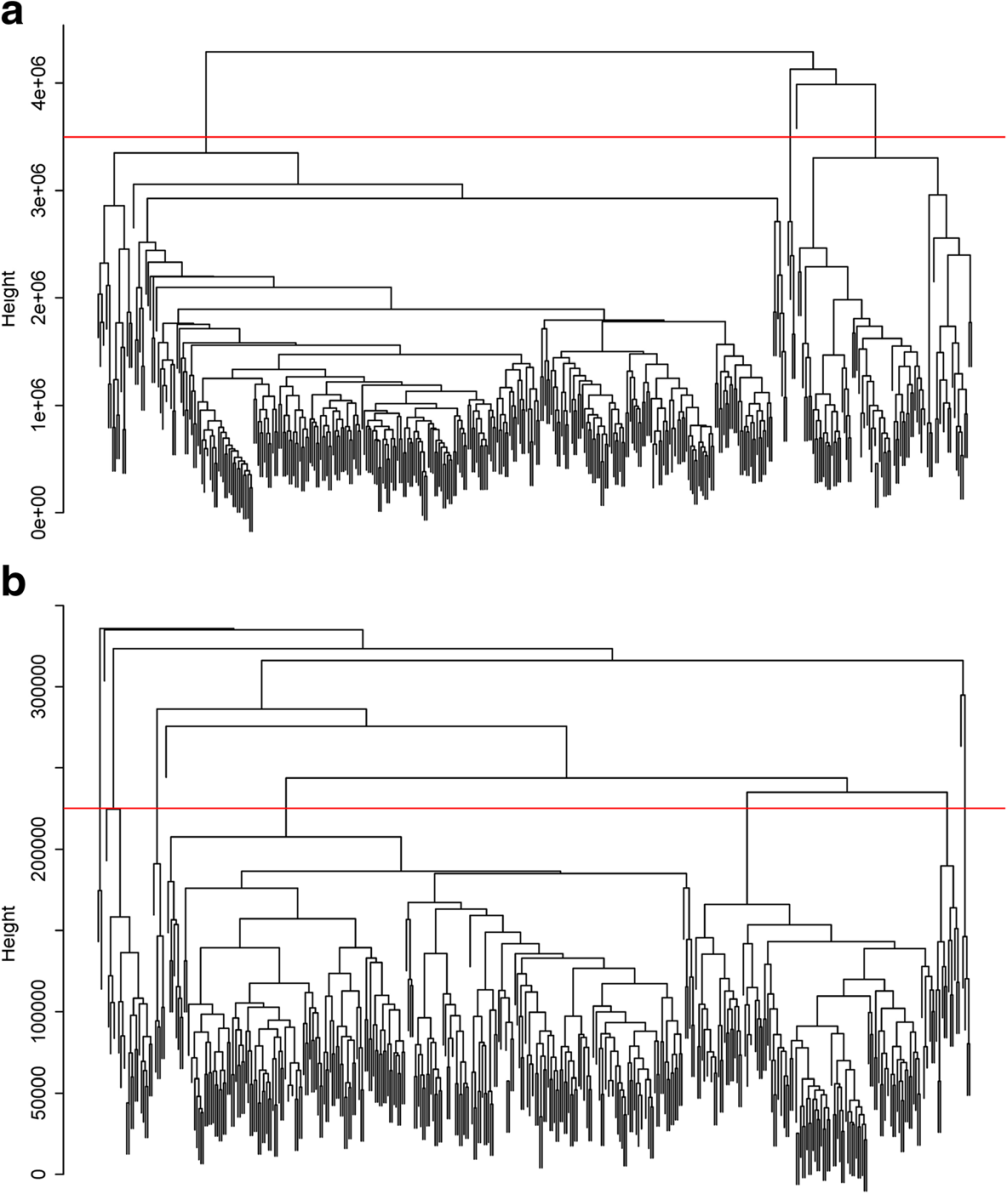
The cluster dendrogram of co-expression network modules for mRNAs (**a**) and miRNAs (**b**). The *red line* represents the cut-off of data filtering in the step of data preprocessing

For the mRNA sequencing data, after data preprocessing, 362 mRNAs were removed due to low expression or data missing, and a total of 20,169 mRNAs were remained for further analysis. Subsequently, hierarchically clustering of mRNA expression data for the 371 patients was carried out (Fig. 1a). Deviation between data of some patient samples and the main cluster was more than 40%, thus, 3.5E + 6 was set as the cut-off criteria. Finally, data of 22 patient samples were removed due to deviation >40% or RPKM <10, thus, mRNA data of 349 patients remained for further analysis.

For the miRNA sequencing data, 854 miRNAs remained after data preprocessing. The hierarchically clustering of miRNA expression data for the 372 patients showed that deviation between data of some patient samples and the main cluster was more than 40%, thus, 2.25E + 5 was set as the cut-off threshold (Fig. 1b). Finally, a total of 320 patient sample data remained for further analysis.

### mRNA expression module analysis of WGCNA

**Fig. 2:**
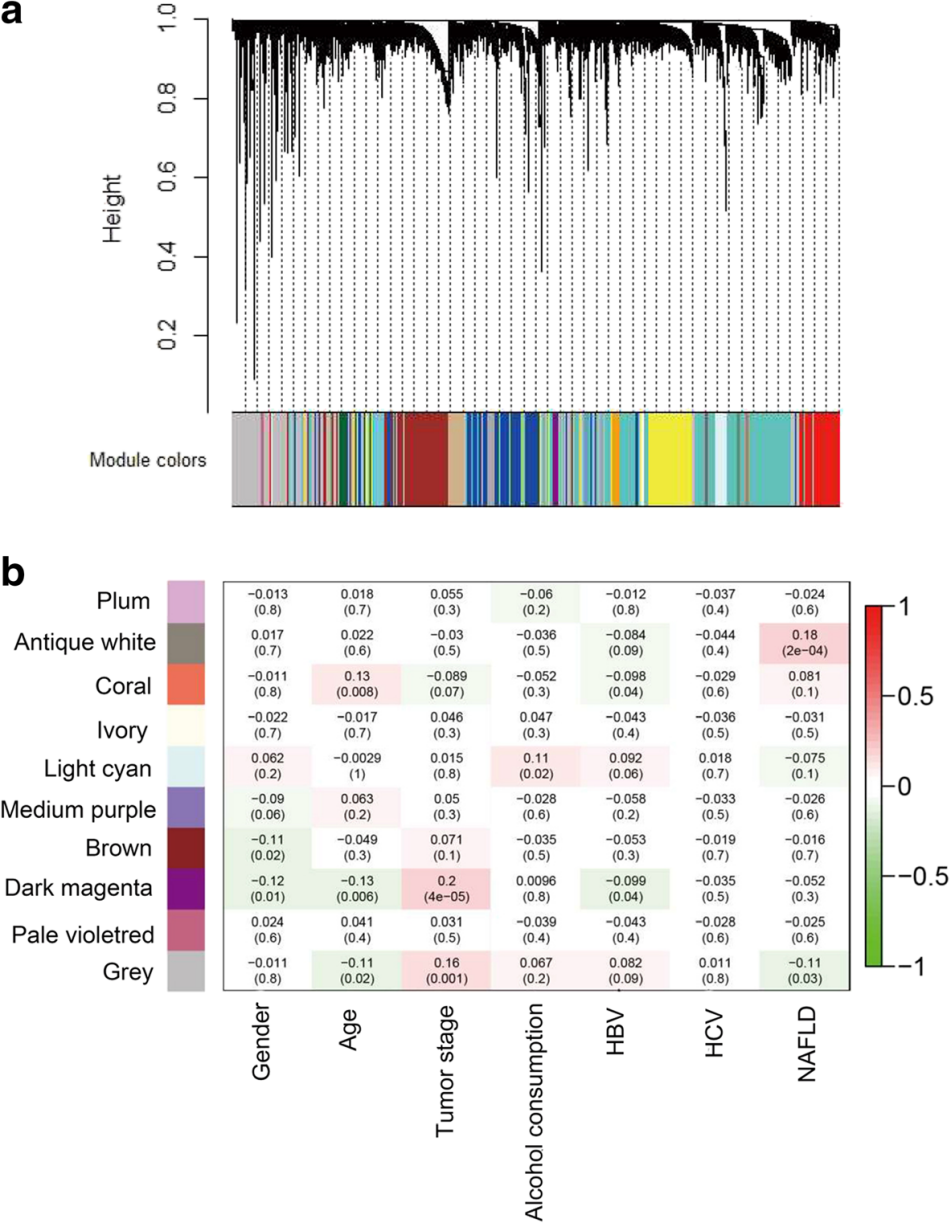
The color display of co-expression network modules for mRNAs (**a**) and the correlation of mRNA co-expression network modules with clinical prognostic factors of hepatocellular carcinoma (**b**). HBV, hepatitis B virus; HCV, hepatitis C virus; NAFLD, non-alcoholic fatty liver disease

In total, 10 mRNA network modules were identified using WGCNA. Because module identification does not make use of prior biological knowledge about the mRNA, the biological meaning of each module is initially unknown and hence the modules were assigned a color label (plum, antique white, coral, ivory, light cyan, medium purple, brown, dark magenta, pale violet and dark grey). (Fig. 2a).

To reveal the functions of the mRNAs in each of the modules, GO enrichment analysis was performed. According to the results, the mRNAs in the dark magenta module (*n* = 8695) were enriched for mRNA that coded for proteins involved in alternative splicing; in the dark grey module (*n* = 6199), they were involved with sensory transduction; in the coral module (*n* = 1488), they were involved with oxidoreductase (Table 1).

**Table 1:** Gene Ontology enrichment analysis of genes in the ten network modules

Module	Gene number	Functional Ontology (top)	*p* value	Discription
Plum	52	keratinization	1.50E-25	Protein involved in keratinization, the process in which the cytoplasm of the outermost cells of the vertebrate epidermis is replaced by keratin. Keratinization occurs in the stratum corneum, feathers, hair, claws, nails, hooves, and horns.
Antique white	122	erythrocyte	1.20E-09	Protein involved in the maturation of erythrocytes, the predominant type of cells present in vertebrate blood and which contain the gas-transporting protein, hemoglobin.
Coral	1488	oxidoreductase	4.20E-49	Enzyme that catalyzes the oxidation of one compound with the reduction of another.
Ivory	124	Secreted	6.20E-11	Protein secreted into the cell surroundings.
Light cyan	3299	acetylation	4.90E-92	Protein which is posttranslationally modified by the attachment of at least one acetyl group; generally at the N-terminus.
Medium purple	35	cell membrane	6.20E-03	Protein found in or associated with the cytoplasmic membrane, a selectively permeable membrane which separates the cytoplasm from its surroundings. Known as the cell inner membrane in prokaryotes with 2 membranes.
Brown	99	glycoprotein	3.90E-04	Protein containing one or more covalently linked carbohydrates of various types, i.e. from monosaccharides to branched polysaccharides, including glycosylphosphatidylinositol (GPI), glycosaminoglycans (GAG).
Dark magenta	8695	alternative splicing	3.20E-39	Protein for which at least two isoforms exist due to distinct pre-mRNA splicing events.
Pale violetred	46	chromosomal protein	1.00E-08	Protein which is associated with chromosomal DNA, including histones, protamines and high mobility group proteins.
Grey	6199	sensory transduction	3.40E-13	Protein involved in sensory transduction, the process by which a cell converts an extracellular signal, such as light, taste, sound, touch or smell, into electric signals.

Correlations between gene network modules and clinical prognostic data were analyzed. Module dark magenta was found to be significantly correlated with tumor stage of HCC (*r* = 0.2, *p* = 4e-5); module antique white was related to NAFLD (*r* = 0.18, *p* = 2e-4); and module coral was associated with age (*r* = 0.13, *p* = 0.008) (Fig. 2b).

Additionally, GO enrichment analysis of the three gene modules that correlated with prognosis of HCC was conducted. The results showed that module dark magenta was significantly related to several functions like alternative splicing and transcription (Table 2).

**Table 2:** Top 5 Gene Ontology functional terms of genes in module dark magenta associated with the clinical prognostic data

Genes numbers	Functional Ontology (top5)	*p* value	Discription
3576	alternative splicing	3.5E-39	Protein for which at least two isoforms exist due to distinct pre-mRNA splicing events.
1038	dna-binding	7.6E-36	Protein which binds to DNA, typically to pack or modify the DNA, or to regulate gene expression.
1091	transcription regulation	2.2E-30	Protein involved in the regulation of the transcription process.
1107	Transcription	3.3E-29	Protein involved in the transfer of genetic information from DNA to messenger RNA (mRNA) by DNA-directed RNA polymerase
2102	nucleus	3.8E-27	Protein located in the nucleus of a cell.

### Weighted Gene co-expression network analysis (WGCNA) of miRNA

**Fig. 3:**
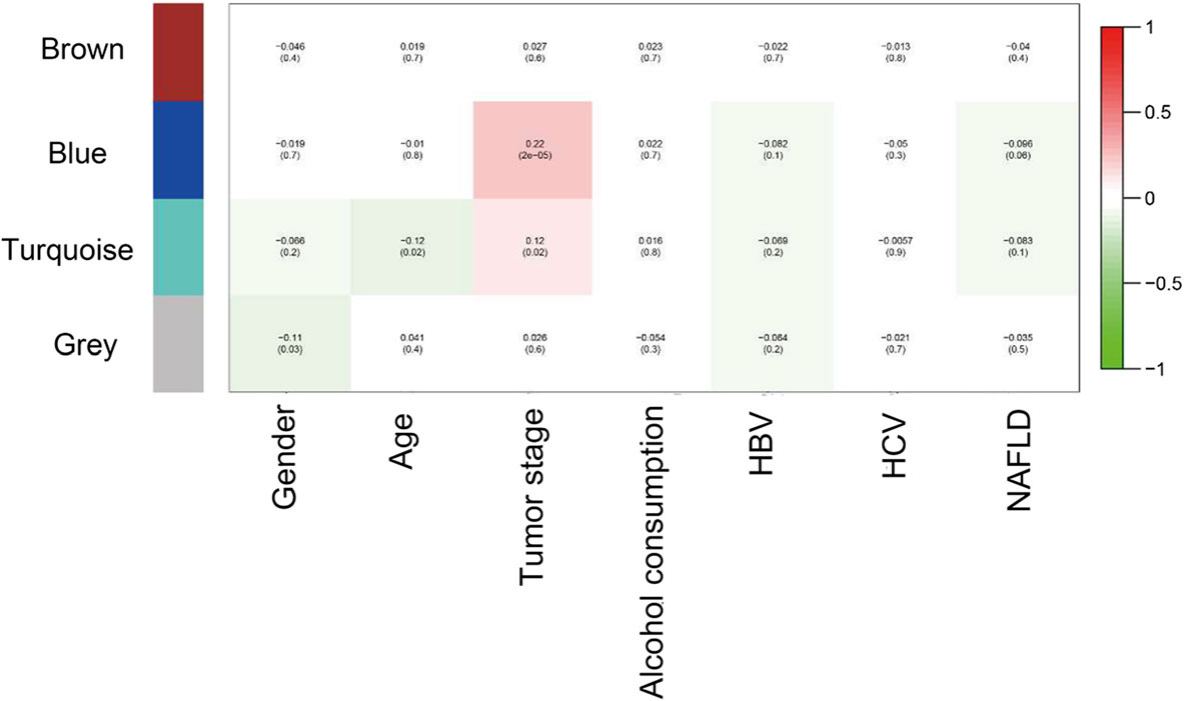
The correlation of microRNA co-expression network modules with clinical prognostic factors of hepatocellular carcinoma. HBV, hepatitis B virus; HCV, hepatitis C virus; NAFLD, non-alcoholic fatty liver disease

According to the module analysis of miRNA co-expression networks, four miRNA network modules were identified, and assigned the color labels brown, blue, turquoise and grey. The correlation analysis of modules and clinical prognostic data revealed that module blue was markedly associated with tumor stage of HCC (*r* = 0.22, *p* = 2e-5) (Fig. 3).

As the most significant module related to tumor stage, 111 miRNAs contained in the module blue were chosen for target genes. A total of 530 target genes were screened out based on the information in TargetScan, miRanda, and miRwalk. These target genes were significantly related to GO functions like alternative splicing, phosphotransferase and immune response (Table 3).

**Table 3:** The results of Gene Ontology enrichment analysis of target genes of microRNAs in module blue

Function ontology	*p* value	Description
Alternative splicing	2.90E-05	Protein for which at least two isoforms exist due to distinct pre-mRNA splicing events.
Phosphotransferase	3.90E-03	Protein involved in the phosphotransferase system, the major carbohydrate transport system in bacteria. This phosphotransferase system catalyzes the transfer of the phosphoryl group from phosphoenolpyruvate to incoming sugar substrates concomitant with their translocation across the cell membrane.
Coiled coil	5.30E-03	Protein which contains at least one coiled coil domain, a type of secondary structure composed of two or more alpha helices which entwine to form a cable structure. In proteins, the helical cables serve a mechanical role in forming stiff bundles of fibres.
Immune response	9.00E-03	Protein involved in immunity, any immune system process that functions in the response of an organism to a potential internal or invasive threat. The vertebrate immune system is formed by the innate immune system (composed of phagocytes, complement, antimicrobial peptides, etc) and by the adaptive immune system which consists of T- and B-lymphocytes.
Transferase	1.40E-02	Enzyme that transfers a chemical group, e.g. a methyl group or a glycosyl group from one compound (donor) to another compound (acceptor).

### Associations of miRNA target genes and gene network modules

**Fig. 4:**
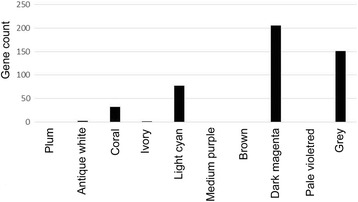
Distribution of microRNA in module blue target genes in the ten gene network modules

To investigate whether miRNA targets in module blue were genes in the gene network modules, distribution of miRNA targets in the 10 gene network modules was analyzed. More than 200 miRNA targets were distributed in the gene module dark magenta (Fig. 4). Among them, the number of target genes of hsa-miR-363-5p in module dark magenta reached 22, including APOBEC3F, ASB16, GAPVD1, IL6R, MRPL44, MTFMT, MYEF2, PPM1D, RASSF2, RELL1, RGPD5, RGPD6, RHOF, RRP15, SHOX, SLC9A7, SPC24, ST8SIA4, WHSC1, ZNF445, ZNF780B, and ZYG11A.

### Correlation of hsa-miR-363-5p and prognosis of HCC

**Fig. 5:**
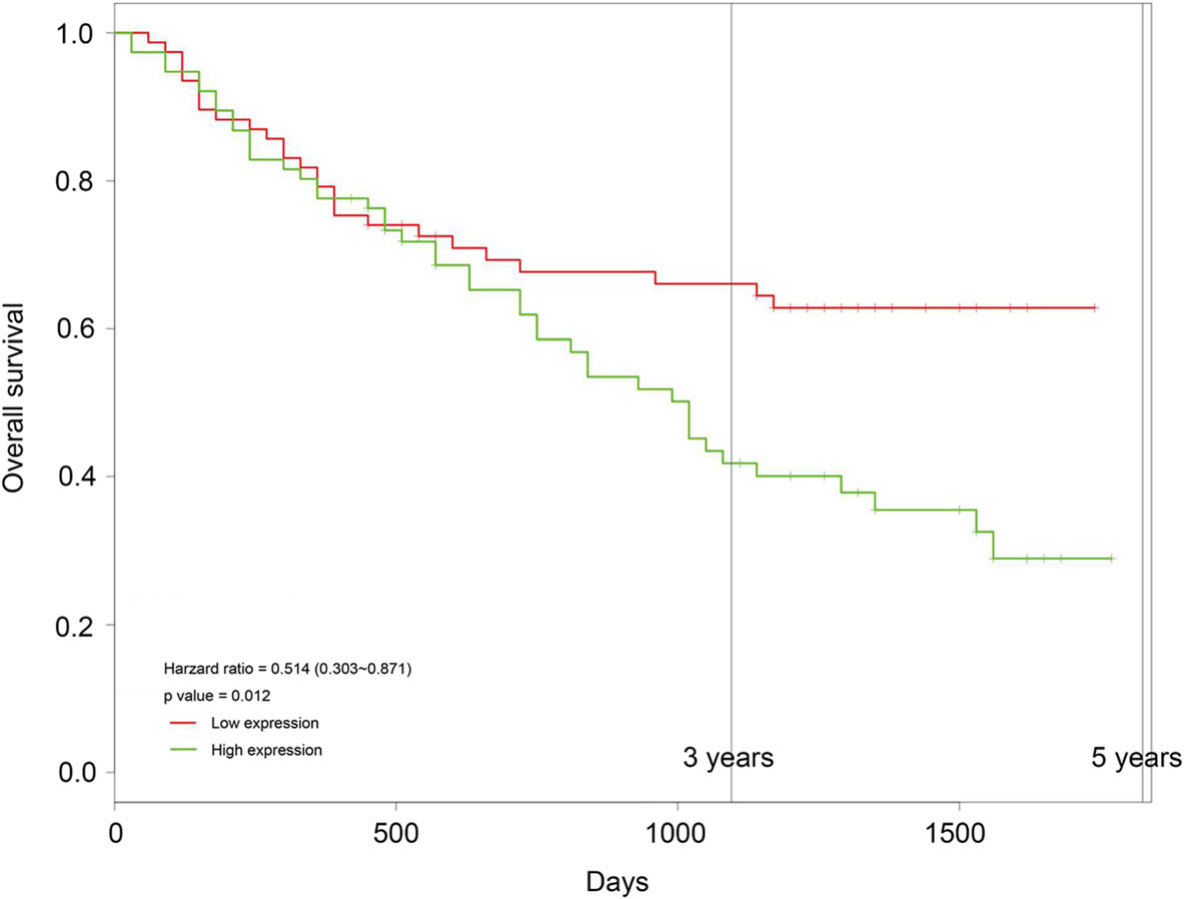
The survival curve displaying the difference of survival rate between the high- and low-expression groups divided by hsa-miR-363-5p expression level

To further reveal the correlation of hsa-miR-363-5p and prognosis of HCC, survival curve was analyzed by Kaplan–Meier test. The median value of hsa-miR-363-5p expression (RPKM = 4.61) was set as the cut-off criteria for high- and low-expression groups of HCC patient samples in TCGA. The 5-year survival curve proved the survival difference between the high- and low-expression groups started at 2 years after surgery, and the difference reached 51.4% 5 years (*p* = 0.012) (Fig. 5) after surgery. The results indicated that hsa-miR-363-5p expression was closely related to the prognosis of HCC.

## Discussion

In the present study, 10 mRNA network modules were identified based on the WGCNA of sequencing data of HCC samples, of which three modules were significantly related to tumor stage, NAFLD and patient age. Meanwhile, four miRNA network modules were identified, of which one module was associated with tumor stage of HCC. Target genes of the miRNAs in this miRNA network module were markedly related to alternative splicing.

Alternative splicing is one of the most significant components of the functional complexity of the human genome, and various splicing alterations have been involved in different steps and aspects of cancer initiation and progression [22]. In HCC, alternative splicing of multiple molecules have been detected. For instance, two alternative splicing isoforms of the cell fate determinant Numb, PRR^L^ and PRR^S^, correlated with the prognosis of patients with HCC [23]. Alternative splicing of A-Raf is regulated by the upregulation of hnRNP A2 in HCC, which activates the Ras-MAPK-ERK pathway [24]. Another study reported that the splicing regulator SLU7 is an essential factor for the preservation of HCC cell viability via oncogenic miR-17-92 cluster expression [25]. Therefore, alternative splicing of some genes plays a critical role in the progression and prognosis of HCC.

Furthermore, a set of target genes of miRNAs were the genes in the gene network modules. Among them, 22 targets of hsa-miR-363-5p were distributed in the gene network modules, such as RGPD5, RGPD6, ZNF445, and ZNF780B. Kaplan–Meier test revealed that low expression of hsa-miR-363-5p was closely related to better overall survival of HCC patients. miR-363-5p belongs to the miR-363 family, and it modulates endothelial cell properties and their communication with hematopoietic precursor cells [25]. Inhibition of miR-363-5p affect angiogenic properties (e.g. the response to stimulation by angiogenic factors) of endothelial cells and the interaction between endothelial cells and hematopoietic precursors [26]. miR-363-5p has been previously predicted to be down-regulated in HCC tumor endothelial cells compared to normal hepatic sinusoidal endothelial cells [27]. Besides, miR-363-5p also exhibits decreased expression in other tumors, such as human oral squamous carcinoma and triple-negative breast cancer [28, 29]. These studies indicated that miR-363-5p is usually downregulated in cancer. However, low expression of hsa-miR-363-5p was found to be closely associated with the better overall survival of HCC patients. There is no report about the association of miR-363-5p with survival of HCC patients to date. We speculate that low expression of miR-363-5p might has effect on angiogenic properties and interactions with hematopoietic precursors, and thus inhibits the angiogenesis in HCC, weakens the invasion ability of tumor and results in a better overall survival of patients. However, this inference is required to be further validated by experiments, which will be conducted in our future study.

A set of genes, such as RGPD5, RGPD6, ZNF445, and ZNF780B were predicted to be regulated by miR-363-5p in this study. Both RGPD5 and RGPD6 share a high degree of sequence identity with RANBP2, a large RAN-binding protein [30]. Currently, there are limited studies about the relations between RGPD5/ RGPD6 and cancer. Both ZNF445 (also known as ZNF168) and ZNF780B (also known as ZNF779) encode zinc finger proteins that may be associated with transcriptional regulation [31]. No evidence is now available to show their connection with HCC. However, a previous study has demonstrated that another member of the ZNF family, ZNF165 mRNA and its protein are expressed in HCC, indicating that ZNF family may be involved in tumor biology of HCC [32]. Besides, a recent study has found that zinc finger protein ZBTB20 promotes tumor growth of HCC via transcriptionally repressing FoxO1 [33]. In our further study, we will confirm the regulatory relationships between miR-363-5p and its target genes (e.g. RGPD5, RGPD6, ZNF445 and ZNF780B), and the associations of these genes with HCC.

## Conclusion

In conclusion, we found that several gene networks and one miRNA network might be important clinical prognostic factors (e.g. tumor stage, NAFLD or age) for HCC based on the WGCNA of sequencing data. Low expression of hsa-miR-363-5p was closely related to the better overall survival of HCC, and it may be a potential prognostic marker for HCC.

## Abbreviations

DAVID: the Database for Annotation, Visualization and Integrated Discovery
FDR: False discovery rate
GO: Gene Ontology
GS: Gene significance
HBV: Chronic hepatitis B virus
HCC: Hepatocellular carcinoma
HCV: Hepatitis C virus
miRNA: microRNA
MS: Module significance
NAFLD: Non-alcoholic fatty liver disease
RPKM: Reads per kilobase of exon per million reads mapped
TCGA: The Cancer Genome Atlas
TOM: Topological overlap matrix
WGCNA: Weighted gene co-expression network analysis
